# Biomedical Approach of Nanotechnology and Biological Risks: A Mini-Review

**DOI:** 10.3390/ijms242316719

**Published:** 2023-11-24

**Authors:** Debora F. Silva, Ailime L. P. Melo, Ana F. C. Uchôa, Graziela M. A. Pereira, Alisson E. F. Alves, Maria C. Vasconcellos, Francisco H. Xavier-Júnior, Marcele F. Passos

**Affiliations:** 1 Technological Development Group in Biopolymers and Biomaterials from the Amazon, Graduate Program in Materials Science and Engineering, Federal University of Para, Ananindeua 67130-660, Brazil; dfsdeborafreitas@gmail.com; 2 Technological Development Group in Biopolymers and Biomaterials from the Amazon, Graduate Program in Biotechnology, Federal University of Para, Belem 66075-110, Brazil; 3 Pharmaceutical Biotechnology Laboratory (BioTecFarm), Department of Pharmaceutical Sciences, Federal University of Paraíba, João Pessoa 58051-900, Brazil; anaflaviauchoauf@gmail.com (A.F.C.U.); fhxj@academico.ufpb.br (F.H.X.-J.); 4 Post-Graduate Program in Bioactive Natural and Synthetic Products, Federal University of Paraíba, João Pessoa 58051-900, Brazil; alissonemannuel@gmail.com; 5 Open Innovation Cosméticos LTDA, Curitiba 80060-100, Brazil; openinnovation66@gmail.com

**Keywords:** nanomaterials, tissue engineering, polymer, controlled drug release

## Abstract

Nanotechnology has played a prominent role in biomedical engineering, offering innovative approaches to numerous treatments. Notable advances have been observed in the development of medical devices, contributing to the advancement of modern medicine. This article briefly discusses key applications of nanotechnology in tissue engineering, controlled drug release systems, biosensors and monitoring, and imaging and diagnosis. The particular emphasis on this theme will result in a better understanding, selection, and technical approach to nanomaterials for biomedical purposes, including biological risks, security, and biocompatibility criteria.

## 1. Introduction

Biomedical engineering combines principles from engineering, biological sciences, and medicine to develop technological solutions in healthcare. With various applications in medical imaging, diagnostic systems, rehabilitation, and more [1,2,3], scientific research has been driving the interest in optimizing advanced medical devices, personalizing medicine, integrating health data, and innovative therapies. For this, a clear intersection with nanotechnology is observed.

**Figure 1:**
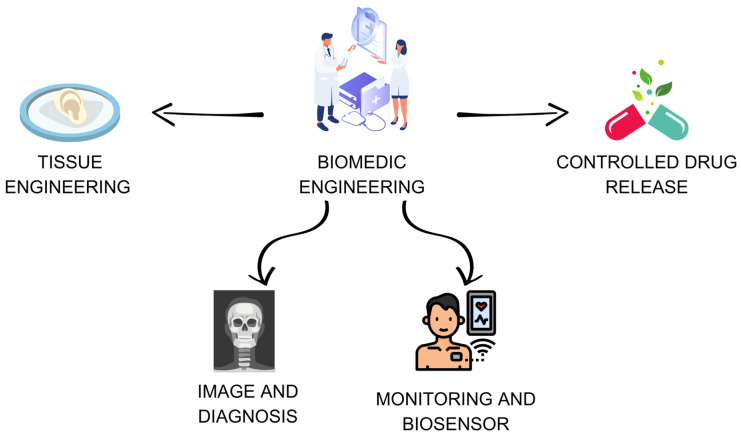
Primary applications of nanotechnology in the field of biomedical engineering.

The use of nanotechnology in the field of biomedical engineering has emerged as a promising approach with the potential to develop more effective treatments and offer a deeper understanding of biological processes at the molecular level. By manipulating materials and devices at the nanoscale, researchers and engineers create structures with exceptional properties and functionalities that remain elusive at larger scales. This remarkable ability to engineer materials and devices at such a precise level has unfolded a vast spectrum of applications in the biomedical field [4,5,6,7], such as controlled drug delivery, biomarker detection, targeted therapies, advanced imaging, biosensors and monitoring, and tissue engineering (Figure 1).

In tissue engineering, one of the most promising areas is the development of artificial blood vessels. Nanomaterials have unique properties due to the surface-to-volume ratio of the structures [8,9,10], which increases the nutrition of cells and the viability of regenerated tissues. Nanotechnological materials such as three-dimensional matrices are suitable for cell growth and differentiation as well as the formation of new tissue. For example, nanofibers, nanoparticles, or nanocomposites can improve tissue adhesion and vascularization by mimicking the extracellular matrix [11,12].

In drug delivery systems, nanoparticles and nanocarriers provide means to encapsulate drugs, vitamins, therapeutics, and particles in general, protecting them from degradation and enabling targeted delivery to specific cells or tissues [4,13,14]. Consequently, this results in increased treatment efficacy and reduced side effects. Additionally, the functionalization of the surface of nanoparticles further increases their specificity for targeted drug delivery [15].

Nanometric surface modifications can improve the biocompatibility of materials, stimulating cell adhesion, preventing bacterial colonization, and modulating immune responses [16,17,18]. The application of these coatings and modifications, based on nanotechnology, promotes a significant improvement in the functionality and overall longevity of implants, ranging from orthopedic devices [19] to cardiovascular stents [20] and dental implants [21].

The application of nanotechnology in diagnostics and imaging in biomedical engineering has also brought about significant changes. Using nanoparticles and nanometric materials can improve imaging methods’ sensitivity, resolution, and detectability, such as magnetic resonance imaging, computed tomography, and fluorescence imaging [22,23,24], allowing for early disease detection, more accurate images, and the real-time monitoring of physiological parameters. In addition, nanotechnology enables the development of highly sensitive and specific nanosensors and nanoprobes to detect biomarkers, greatly expanding diagnostic possibilities [25].

In general, nanotechnology and biomedical engineering are interdisciplinary fields with significant impacts on health. The design, maintenance, and development of devices, including the manipulation of matter at the nanoscale, bring innovative therapeutic approaches and advances in applications. The use of nanomaterials in tissue engineering, drug delivery, diagnostics, imaging, and surface modifications has opened new pathways for personalized medicine and targeted therapies, reducing side effects and damage to healthy tissues. This mini-review presents an overview of the most recent advances in nanotechnology and its application in biomedical engineering (drug delivery, imaging, and diagnosis, etc.) as well as specific information on nanomaterials in tissue engineering through the development of nanoemulsions, nanowires, and carbon nanotubes. Electrospinning and chemical vapor deposition were presented as potential technological strategies for manufacturing nanomaterials. Mechanisms of nanomaterials in the intracellular environment were also discussed with an analysis of their biological risks and the need to study biocompatibility.

## 2. Nanomaterials and Tissue Engineering

Nanotechnology is a multidisciplinary field of science and engineering that deals with designing, synthesizing, manipulating, and applying materials and devices with at least one dimension at the nanoscale [26], which makes their physical and chemical properties different from the macroscale, producing unique properties and substantial benefits [27]. Still, as a challenging and growing field, it has potential in several segments. Nanotechnology offers new opportunities for controlled drug release in health with drug delivery directly to specific cells or tissues [28]. And it has allowed the creation of new implants and prostheses with significant advances in medicine and regenerative tissue engineering [29,30].

Tissue engineering creates solutions to regenerate, replace, or repair damaged tissues or organs. Biomaterials, cells, and growth factors are important in this area. Through these, it is possible to develop designed structures and a controlled environment for cell culture and differentiation, aiming to form a new tissue [31]. The structures, known as scaffolds, play an important role, acting as a physical, chemical, and mechanical support for cells, guiding cell growth [32]. Some of their fundamental characteristics are their three-dimensional structure, porosity and interconnectivity, controlled degradability, surface suitable for cell adhesion, and biocompatibility [33]. Depending on the application, scaffolds can be manufactured from different biomaterials (natural, synthetic, hybrid, composite, etc.), designed with other technologies, or chemically modified on the surface to adapt to the application (bone regeneration, cartilage, skin, muscle, etc.) or increase the efficacy and functioning of implants [34]. Thus, continuous technological advances bring new strategies for manufacturing scaffolds as environments conducive to forming functional tissues, driving innovations in regenerative medicine and health. A fundamental advantage of nanotechnology in tissue engineering lies in its ability to design materials with customized mechanical and biological characteristics similar to those found in natural tissues [27]. However, it is crucial to ensure the human biosafety assessment of nanomaterial products before using them in clinical settings. To date, most available data concerning nanomaterial products in nanomedicine are based on in vitro cell culture or in vivo experiments with animals. For nanomaterial products to be adopted clinically, human clinical trials are the most important stage. Nevertheless, since clinical translation in nanomedicine is a protracted, challenging, and resource-intensive process, it is unlikely that a successful transition will occur, as emphasized by Satalkar et al. (2016) [35].

### 2.1. Nanoparticles and Nanowires

Nanoparticles are one of the most used types in tissue engineering, as they provide the controlled release of bioactive growth factors and adhesion molecules while also improving cell adhesion and viability, stimulating the regeneration of damaged tissues, or promoting the formation of new tissues [36].

For the direction and delivery of stem cells, nanoparticles are designed to act as transport vehicles, carrying the stem cells to the target site. The modulation of endogenous adult stem cell niches has been explored for tissue regeneration and the treatment of cellular abnormalities [37]. The dynamics of stem cell differentiation after exposure to nanoparticle formulations were investigated, providing valuable insights into the modulation of the stem cell niche [38]. The authors demonstrated that the release of retinoic acid from polymeric nanoparticles promotes the differentiation of neural stem cells in vivo through interactions with the retinoic acid receptor and activation of signaling pathways. 

Nanoparticles can also monitor and modulate cellular interactions during regenerative therapy. Nanometric sensors can be incorporated into nanoparticles to monitor cellular activity, immune response, and levels of relevant biomarkers [39,40], allowing the real-time monitoring of therapy progress and adapting treatment strategies if necessary [41].

On the other hand, nanowires have a filamentary structure, with a solid and continuous shape, and generally refer to electrically conductive materials. Their conductive potential allows them to emerge as enabling tools for electronic communication with biological systems, opening new perspectives for bioelectronic devices [42,43,44] and playing a crucial role in the interface between nanotechnology and tissue engineering. As scaffolds, they can provide electrical signals to cells, promoting cell communication. If functionalized with bioactive molecules, they can be used in controlled release systems and applied in tissue engineering for organs such as the liver, heart, and bone [45,46]. In addition, conductive nanowires are interesting for regenerating nervous and muscular tissues by creating electrochemical interfaces with cells [47]. As biomimetic sensors, they can monitor the concentration of biomarkers and cell activity, among others, being of paramount importance for studies of tissues in vitro or in vivo [48,49].

### 2.2. Nanofibers

Nanofibers are elongated and thin structural units characterized by their reduced dimensions (1 to 10 nm) and significant length. They are typically made of polymers and proteins and can interact at nanometric scales with complex biological systems [50]. One of the main advantages of their use is the flexibility to adjust the chemical composition, size, and morphology, which directly impact their physicochemical and biological properties [51,52]; this provides a vast field of possibilities in applications such as tissue regeneration, the controlled release of therapeutic agents, and cellular targeting [53,54,55].

In tissue engineering, nanofibers are capable of guiding cell growth [51] and providing adequate structural support for cells [56], promoting adhesion, proliferation, and cellular differentiation [57] as well as allowing the diffusion of nutrients and the elimination of waste. As scaffolds, they are interesting materials [58] due to their high surface area, porosity, surface, and biomimetic characteristics, replicating the extracellular matrix’s structure. Still, they can have modulable mechanical and chemical properties and functionalization with growth factors and signaling molecules, among other properties [59]. In addition, they can mimic the structure and organization of collagen fibers in tissues such as skin, bones, and cartilage [60], establishing a platform for the regeneration of complex tissues.

One of the techniques for producing these nanomaterials as scaffolds is electrospinning, which allows the controlled production of nanofibers in terms of size and composition. In this method, the application of electric fields causes the extrusion of polymer solutions, resulting in the formation of nanofibers with precisely controlled dimensions [61]. The evolution of chemical vapor deposition (CVD) methodologies can also be addressed, highlighting the potential for production with advanced levels of precision. That is, the deposition of materials at the atomic scale results in structures with exquisite accuracy and uniformity [62]. However, there are still challenges to overcome with nanofibers as scaffolds, such as the production scale, fiber uniformity, and use of toxic solvents, depending on the manufacturing technique. Modifications and functionalization on the surface of nanofibers are, therefore, decisive in improving cell interactions, adhesion, proliferation, and differentiation.

The perspective of customization through surface modifications, exemplified by Mozaffari et al. (2021) [63], emerges as a highlight. The ability of targeted functionalization to enhance interaction with target cells or confer specific properties is possible for nanofibers. One approach is chemically modifying the surface by linking functional groups that mimic extracellular matrix components or have an affinity for specific cells [64]. These modifications can include the introduction of bioactive molecules, such as proteins, peptides, or growth factors, that promote targeted cellular interactions and appropriate signaling for tissue development and regeneration [65]. A study investigated the modification of biodegradable nanofibers by plasma to improve the adhesion and proliferation of mesenchymal stem cells (MSCs) [66]. The fibers with a high CO_2_:C_2_H_4_ ratio showed a well-defined actin microfilament network in the MSCs, while the threads with a low CO_2_:C_2_H_4_ ratio showed inferior cell adhesion and survival. These results highlighted Ar/CO_2_/C_2_H_4_ plasma polymerization as a promising tool for modulating MSC viability.

Composite scaffolds of E7 peptide and silk fibroin modified with polydopamine showed improved hydrophilicity, proliferation, and cell adhesion as well as an increased osteogenic differentiation of bone marrow mesenchymal stem cells (BMSCs) [67]. Therefore, by modifying the surface of scaffolds or biomaterials, it is possible to improve cell interactions, promoting the adhesion, proliferation, and differentiation of cells relevant to the tissue under regeneration. In addition, surface modification can increase the biocompatibility of biomaterials, reduce the immune response, and improve integration with the surrounding tissue [65].

### 2.3. Nanoemulsions

Nanoemulsions are nanomaterials characterized by their small droplet size and colloidal stability. Composed of immiscible liquids, such as oil and water, along with an emulsifier, they have droplets in the range of 20 to 200 nanometers [68], and their general characteristics resonate within biomedical and tissue engineering [69].

Understanding the characteristics of nanoemulsions interferes with the final configurations of the nanometric architecture. Changes in pH, temperature, or other conditions can trigger changes in the properties of nanoemulsions, allowing adaptive and programmed responses [70]. Precision in controlling particle size, size distribution, composition, and stability is vital for functionalizing their intrinsic properties [71]. The reduced size of the particles is one of the most distinctive characteristics. These dimensions allow for homogeneous dispersion and a high surface area, which are essential for bioactivity interactions [72].

Stability is influenced by the choice of emulsifiers, which act as maintenance agents between the phases, preventing any unwanted coalescence of the particles. This stability guarantees a prolonged shelf life and consistent long-term efficacy [73]. In addition, nanoemulsions offer compositional versatility, encompassing the study of hydrophobic and hydrophilic substances. There is the flexibility to incorporate a variety of active ingredients, such as drugs and therapeutic agents [74]. These combined characteristics make controlled and targeted release more feasible, allowing the properties of nanoemulsions to be adjusted to release bioactive substances gradually and precisely at the desired target [75].

The formation of nanoemulsions requires specific knowledge and skills involving high-energy processes, such as ultrasound, cavitation devices, or high-pressure homogenization [76,77,78], which break larger droplets into smaller droplets, resulting in a stable emulsion [79]. It also requires low-energy methods, such as spontaneous emulsification and phase inversion. In the low-energy emulsification method, a gentle agitation is used to break the particles, which is usually applied to sensitive substances [80]. Kumar et al. (2021) [81] obtained a nanoemulsion system for drug delivery by phase inversion. This method explores the change in affinity of lipophilic and hydrophilic components in response to changes in conditions, such as pH or temperature. However, it is also possible to obtain nanoemulsions from other methods, such as complex coacervation that uses polymers and surfactant agents to form coacervates [82]. Surface modifications of nanoemulsions, with coating polymers, are still exciting approaches to personalize them with specific ligands for applications in various biomedical fields.

Nanoemulsions have the potential for diagnostic applications. Their ability to carry contrast agents, such as in medical imaging exams, expands the boundaries of precise diagnosis [83]. Joga et al. (2022) [84] describe the use of various functionalized oil-in-water nanoemulsions as pharmacological vehicles with potential for theranostics in cancer treatment, incorporating components such as vitamin E, oleic acid, sphingomyelin, ligands for functionalization, contrast agents, and therapeutic biomolecules, and the results showed the adequate physical stability of the formulation.

By incorporating these substances into nanoemulsions, it is possible to optimize the regeneration of complex tissues, directing their transport to specific parts of the body and controlling the release rate. Systems of α-tocopherol encapsulated in curcumin nanoemulsions showed improved collagen deposition and prevented bacterial contamination, accelerating wound healing in diabetic animals [85].

Nanoemulsion delivery systems can also carry various bioactive compounds, such as vitamins and even probiotics, to incorporate them into food [86]. Similarly, gel systems of nanoemulsions loaded with resveratrol can act on UV-induced oxidative damage in the skin [87].

### 2.4. Nanotubes

Nanotubes are cylindrical hollow nanostructures characterized by diameters that vary in the nanometer range and have exceptional potential to positively influence a wide range of biomedical applications. Especially in tissue engineering, nanotubes emerge as versatile and multifunctional components capable of addressing complex challenges. Their intrinsic characteristics offer the ability for precise interaction at nanometric scales and have revealed unique properties that open up avenues for innovations in diagnostics [88], therapeutics [89], and tissue regeneration [90]. In addition, nanotubes have influenced cell adhesion, proliferation, and differentiation. Their unique morphology and modifiable surface enable the creation of three-dimensional scaffolds capable of guiding cell growth and promoting the formation of functional tissues [91].

Carbon nanotubes reveal a fascinating structural diversity, presenting chiral, armchair, or zigzag arrangements [92]. These unique structures give them chemically inert properties, resisting unwanted chemical reactions and preserving their integrity when interacting with complex biological environments [93]. Additionally, the singularity of their chirality stands out, as it influences their intrinsic electronic and mechanical properties [94], and the stability of these properties is remarkable, allowing reliable performance even in challenging conditions [95]. In addition, the number of graphene walls and the diameter of these nanotubes emerge as crucial factors, directly shaping their conductivity [96]. This fine tuning between structure and functionality makes them ideal building blocks for nanoelectromechanical sensors (NEMSs), enabling the precise detection of mechanical phenomena, such as displacements and vibrations in biological systems [97].

The high tensile strength of carbon nanotubes, combined with their exceptional surface-to-volume ratio, also confers robustness to them at the nanoscale [98]. This unique combination of characteristics culminates in a tremendous electrocatalytic activity, providing a versatile platform for complex biochemical reactions [99] and becomes an essential tool in biomedical engineering. Thus, the hollow structure of some nanotubes allows for the controlled release of growth factors, optimizing the regeneration process [100], and this can be applied from detecting biomolecules to creating high-sensitivity devices for precise monitoring and diagnosis.

Due to their large surface area and accessibility to the internal area of the tube, carbon nanotubes are a highly suitable platform for the loading and controlled delivery of bioactive molecules [101]. In addition, they can be functionalized with specific ligands, allowing the creation of highly selective delivery systems capable of interacting with specific biological targets [102]. By designing specific immobilization surfaces for the enzyme glucose oxidase, nanotubes become highly sensitive and selective measurement tools capable of quantifying glucose levels in various body fluids [103]. This innovative approach overcomes the limitations of traditional monitoring techniques, offering more accurate and faster detection of glucose level variations. Also, the intrinsic ability of nanotubes to conduct electricity provides a promising platform for the creation of advanced biosensors, enabling the multiplex detection of multiple biomolecules simultaneously [104].

As for nanotube production techniques, they have evolved considerably to meet the demands of biomedical engineering. Chemical vapor deposition (CVD) synthesis and anodization are notable examples. The former involves material deposition on substrates by chemical reactions in the gas phase, making it possible to obtain nanotubes with precision and control [105]. Anodization, on the other hand, is an electrochemical method that allows the controlled formation of nanotubes on metal surfaces, offering a versatile approach to creating nanometric structures [106].

## 3. Nanomaterials in Biosensing and Monitoring

The field of biosensors and monitoring has made significant advances, using precise, reliable, and portable tools to track processes or detect biological substances in real time and remotely. Nanomaterials have been widely used for this purpose, aiming to identify and quantify biological molecules, pathogens, and other species of interest [107]. Thus, integrating nanotechnology with the biosensing system gives rise to nano-biosensors [108] with specific surface and chemical attributes. Their advantages include flexibility, rapid detection, accuracy, reproducibility, versatility, and high electrical conductivity [109]. In addition, nano-biosensors improve the immobilization of recognizable molecules (adsorption, microencapsulation, entrapment, covalent bonding, and cross-linking) [110].

**Figure 2:**
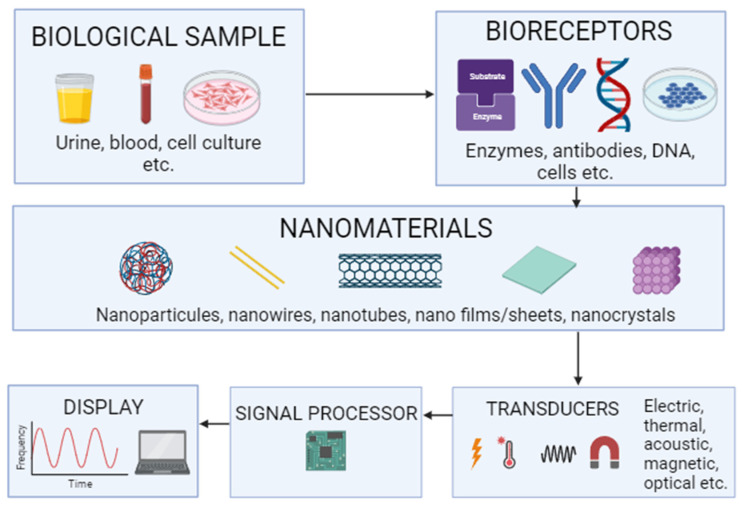
Components of a nano-biosensor.

Nano-biosensors, combined with biological elements (enzymes, antibodies, and nucleic acids) and sensitive detection platforms, convert biomolecular interactions into measurable signals [111]. They can be applied in biomedical engineering as medical diagnostic devices. In general, nano-biosensors can be classified based on their material components, the specific targets they detect, and the signals they use to transmit information [112]. Bioreceptors and transducers are their main components (Figure 2). Bioreceptors comprise biomolecules that detect biochemical interactions or the analyte [113]. The transmission of the biological signal as an electrical signal is carried out by the transducer, which can be calorimetric, potentiometric, amperometric, or optical, among others [114]. More specifically, biochemical signals are converted into electrical signals due to the interaction of the bioreceptor with the analyte [115].

### Applications of Nano-Biosensors in Healthcare

The combination of nanotechnology with biosensors has allowed for a new type of biomolecular analysis with high sensitivity [110], remote monitoring, and compact and small-scale manufacturing, which has led to a new range of possibilities in the environmental and health field, such as the improvement of diagnostics, imaging, monitoring, detection, and regenerative medicine [116]. Regarding energy, the nanosensors can still be sustainable [117]. Shu et al. (2020) [118] developed lead and silver sulfide fluorescence quantum dots as H_2_O_2_ biosensors. With high sensitivity and selective potential, the nanomaterial can result in smaller, longer-lasting batteries, reducing the disposal of electrochemical batteries.

Implantable, compact, and flexible nanosensors also have been used to monitor vital signs [119]. Electrical, acoustic, optical, or magnetic signals send real-time measurements to a monitoring system [120], which is essential for helping patients who need continuous monitoring to detect risk factors. In addition, these devices can provide comfort and remote monitoring [121]. In the case of some types of cancer (breast, lung, prostate, and others), nano-biosensors can enable early diagnosis [122] so that treatment can be carried out with the highest possible efficiency and efficacy. Diagnosis can be made by detecting circulating tumor cells and specific biomarkers [112].

When detecting blood glucose to assess diabetes, it is also possible to use nano biosensors. In this sense, some devices are made of glucose oxidase enzymes, which are divided into three generations: (1) glucose detection by the reduction in the consumption of hydrogen peroxide as a substrate; (2) detection through redox mediators with specificity and selectivity to the oxygen present in the medium; and (3) direct electron exchange at the electrode with enzymes [123].

One of the nanomaterials used is graphene. It is interesting because it has a large surface area-to-volume ratio, performs satisfactorily with other functional groups, and has good electron transfer. In addition, it is also low cost [114], representing an adequate alternative to detecting and monitoring blood sugar levels in patients with diabetes.

For detecting SARS-CoV-2, it is possible to use biomarkers in association. The ideal device for this application involves high sensitivity, selectivity, rapid real-time response, and easy use [124].

A study demonstrated the use of nanosensors for detecting the virus in clinical samples, where the sensor was fabricated with graphene films equipped with the specific antigen of the SARS-CoV-2 S protein [125]. However, there is a limitation associated with this method, where the selectivity, accuracy, and reliability of these tests are in question due to the possibility of cross-reaction of the antibodies used, which could lead to false positives [126].

In tissue engineering, nano-biosensors can be used to monitor the performance of artificial tissues and their biological interactions. In the case of wound healing, some biomarkers are essential to regulate the increase in cytokines [127]. Moreover, nano-biosensors can help detect such factors, preventing wound healing from occurring unsuccessfully. In addition, nano-biosensors can determine cellular metabolic activity (signals, responses, behavior, and functionalities) [128]. More specifically, these devices can detect glucose inefficiency by measuring it in the blood and understanding stages of the wound-healing process, such as inflammation or infection [129].

## 4. Delivery Drug

**Figure 3:**
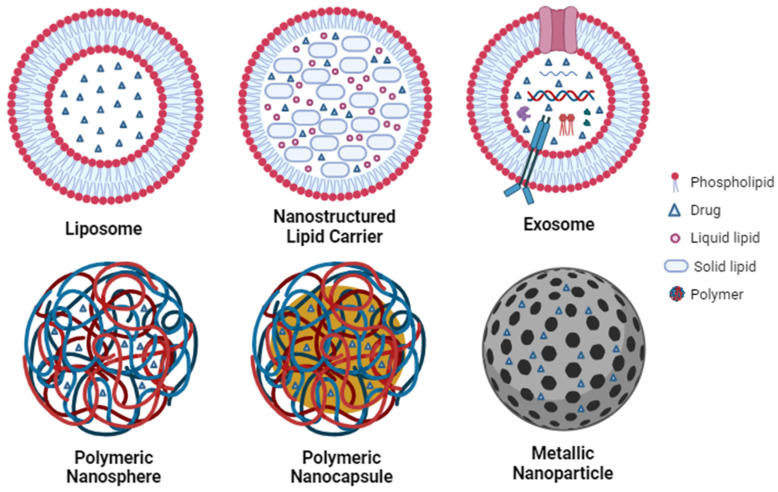
Structure of the main nanoparticles used as drug delivery systems.

Nanotechnology can be used for drug delivery systems to vectorize drugs or other bioactive compounds directed to specific cellular or molecular targets. Within the various nanosystems designed for encapsulating bioactive compounds and facilitating drug delivery, noteworthy examples are lipid nanoparticles, such as liposomes and exosomes, nanostructured lipid carriers, polymeric nanoparticles, such as polymeric nanocapsules and nanospheres, and metallic nanoparticles, such as iron, gold and silver nanoparticles (Figure 3). The small particle size associated with using biodegradable materials makes nanoparticles a great delivery vehicle since they can significantly increase the solubility and bioavailability of the actives in biological fluids, facilitating drug uptake by cells and penetration through biological barriers [130,131,132].

### 4.1. Lipid Nanoparticles

#### 4.1.1. Liposomes

Liposomes are spherical particles composed of phospholipids arranged in a lipid bilayer to form spherical vesicles encapsulating an aqueous core. Depending on the production method, these particles can be obtained on a nanometric scale—from 50 nanometers—or a micrometric scale [133]. If the bioactive compound is hydrophilic, it will be encapsulated within the aqueous core; if it is a lipophilic agent, it will be dispersed in the lipid bilayer [134]. Because they are mostly composed of lipids, their main advantages are biocompatibility and biodegradability. In addition, liposomes are well-known and studied systems; they are still the most used vehicles for drug delivery, either encapsulating drugs or transporting DNA or proteins. Despite this, liposomes have mostly low encapsulation capacity compared to other nanosystems. They cannot release the drug at the desired reduced rate, in addition to being unable to penetrate cells effectively, releasing its contents only into the extracellular environment [135,136].

To significantly prolong the period of permanence of liposomes in the bloodstream and inhibit their absorption by the reticuloendothelial system, their surface can be coated with polyethylene glycol (PEG). Desired drugs can also be incorporated into the aqueous phase of liposomes using an ammonium sulfate gradient to counterbalance unwanted rapid release, allowing for more efficient encapsulation and minimal drug loss into the bloodstream [137].

The liposomes’ physical–chemical characteristics can help them penetrate specific biological barriers; their surface can also be manipulated to reach the desired organs or tissues by modifying the charge, the shape of the particle, or the ligands conjugated. Methods have been developed, for example, to make liposomes hydrophobic or create electrostatic charges, such as magnetic cationic liposomes [138], which allows fast and improved gene delivery to epithelial THLE-3 cells in the liver.

PEG-coated liposomal doxorubicin (Doxil^®^) was the first nanomedicine approved by the Food and Drug Administration (FDA), being used to improve the treatment of breast cancer, increasing the effective concentration of the drug without enhancing the total dose of the medicine (avoiding more side effects), and decreasing cardiotoxic effects [134,139]. Gemcitabine, another chemotherapeutic, was also incorporated into unilamellar liposomes coated with PEG, showing higher rates of apoptosis and inhibition of cell proliferation compared to the free drug [140].

Another drug successfully encapsulated in liposomes is dexamethasone, which is widely used to treat various diseases. Liposomal dexamethasone was effective against an advanced model of myeloma, remaining for a prolonged time in the bloodstream, whereas free dexamethasone was ineffective at the same dosage [141]. In addition, liposomal carfilzomib presented greater efficacy and less systemic toxicity for the same type of tumor. A combined drug therapy generally tends to have a synergistic therapeutic action in the body. A combination of liposomal carfilzomib with doxorubicin was also developed, which resulted in a synergistic action superior to that of the combination of the same two drugs in their free forms [142,143].

Still, within the scope of anticancer activity, Franca et al. (2022) [144] observed that liposomes could be used to deliver β-lapachone to cancer cells. For this purpose, liposomes coated with Concanavalin A were designed, which were more internalized by MCF-7 cells, being considered adequate for treating breast cancer. In the study by Lotfabadi et al. (2018) [145], a novel formulation of cationic liposomes loaded with miRNA was prepared to act against bone marrow cancer cells. The liposomes produced showed cytotoxicity approximately 12% higher than pure miRNA-101 in these cells, reduced toxicity in healthy cells and could be considered a novel gene therapy system.

Regarding cardiovascular health applications, drug delivery through liposomes is effective, especially in preventing platelet aggregation, atherosclerosis, and thrombosis. The administration of a liposome system carrying prostaglandin E-1 (PGE-1) (Liprostin™) has been undergoing clinical trials to improve the treatment of various cardiovascular diseases, such as restenosis after angioplasty, as PGE-1 induces vasodilation, inhibits platelet aggregation and decreases inflammation. In addition, liposomes of cyclic arginyl-glycyl-aspartic acid peptide (cRGD) encapsulated with the thrombolytic drug urokinase have been studied to generate a more selective binding to GPIIb/IIIa receptors, which has been shown to improve the thrombolytic efficacy of the drug by almost four times compared to its free form [146].

Liposomes loaded with therapeutic agents can effectively reach and remain in the ischemic myocardium, especially if coated with PEG. In this regard, Hwang et al. (2016) [147] sought to determine whether PEGylated liposomes effectively improve the treatment of myocardial ischemia. It was seen that the uptake of liposomes was significantly greater when they were 100 nm rather than 600 nm in diameter, with the addition of PEG significantly increasing the myocardial uptake of liposomes. These nanoparticles loaded with angiogenic peptides improved myocardial perfusion defects and increased vascular density. Silica hybrid liposomes were produced by Lee et al. (2018) [148] for the controlled release of Citrus unshiu extract. After 10 h, the silicified liposomes were able to retain 89% of the extract and obtained good release kinetics. The maximum release of the controlled release profile is 41.4%, being considered effective antioxidants.

Liposomes modified with pyrrolidinium surfactants containing a hydroxyethyl fragment have also been prepared for the transdermal delivery of non-steroidal anti-inflammatory drugs. Kuznetsova et al. (2021) [149] reported an encapsulation efficiency of ketoprofen and meloxicam ranging from 75 to 99% and ex vivo transdermal diffusion with a total amount passed through the skin during 51 h from 140 to 162 μg/cm^2^. Resveratrol was also encapsulated by Lafarge et al. (2022) [150]; its transdermal passage in excised human skin drastically increased with encapsulation (about 73% after ten hours of incubation). While the free drug underwent cis isomerization, the liposomes protected it for up to 9 h before undergoing any chemical changes.

Cationic lipid-based transfection reagents, such as lipofectamine, are widely used to transfect cultured cells in vitro. However, they are incompetent for in vivo use due to the high toxicity of cationic lipids and low transfection efficiency of in vivo tissues because of a massive interaction with anionic cellular membranes. Therefore, there have been many attempts to modify the lipid-based vectors to provide safer and greater efficiency [151].

#### 4.1.2. Exosomes

Exosomes are a subgroup of nanometric extracellular vesicles enveloped by a lipid bilayer membrane and secreted by most eukaryotic cells. They represent an intercellular communication route and participate in a wide variety of physiological and pathological processes. The biological functions of exosomes depend on their bioactive cargoes, including proteins, nucleic acids, and lipids, which are delivered to target cells. As they possess high stability, low immunogenicity, biocompatibility, and good biomembrane penetration ability, they function as natural nanocarriers [152].

They stand out due to their efficient potential to deliver molecules to cells through direct membrane fusion, receptor-mediated endocytosis, macropinocytosis, or phagocytosis [153]. Its highly asymmetric lipid bilayer may be advantageous for interacting with target cells [152]. These properties led researchers to explore its usefulness as a drug delivery vehicle for the treatment of a variety of diseases, especially cancer, since tumor cell-derived exosomes have surface receptors similar to tumor cells, which facilitates the entry of these drug-containing nanoparticles into tumor cells [154].

Furthermore, the use of exosomes from other living beings, such as cattle, has been highlighted on the world stage. Studies prove that bovine milk can be a scalable source of exosomes, acting as carriers for chemotherapeutic or chemopreventive agents. Drug-loaded exosomes showed significantly greater efficacy than free drugs in cell culture studies and against lung tumor xenografts in vivo. Tumor-targeting ligands, such as folate, also increase the targeting of exosomes to cancer cells, resulting in increased tumor shrinkage [155].

The production of exosomes is not limited to the animal kingdom either, as these particles were first discovered in fungi and plants. As exosomes are products of living cells, there is always a risk of changing the composition and/or content of these vesicles when they are produced in an artificial environment [156].

Exosomes, combined with their inherent loading capacity and versatility, can be engineered to specifically target cancer cells, thereby minimizing off-target effects and increasing the effectiveness of cancer therapy. Exosomal formulations attenuated the toxic effects of various drugs in murine cancer models [157]. To make them a viable nanodispersion and drug delivery platform for cancer therapy, they must be isolated in large quantities and with a high degree of purity. Exosomes derived from U-87 glioma cells can be used for the administration of paclitaxel, significantly increasing its cytotoxicity and being considered a suitable system for the administration of drugs for the treatment of glioblastoma [158].

Another promising application for the use of exosomes is in the treatment of type 1 diabetes mellitus. Nojehdehi et al. (2018) [159] proved that exosomes derived from adipose tissue-derived mesenchymal stem cells have immunomodulatory effects on the inflammatory response of T cells. Thus, they promote the reduction in clinical symptoms in induced type 1 diabetes mellitus by streptozotocin, having a significant increase in the levels of interleukins (IL-4, IL-10) and a decrease in the levels of IL-17 and interferon-γ in agreement with the significant increase in Treg to cell proportion in splenic MNCs.

Alzheimer’s disease can also potentially be treated with the help of nanotechnology since there is still a lack of effective therapeutic approaches due to the inability of common drugs to cross the blood–brain barrier. Wang et al. (2019) [160] produced exosomes containing curcumin to prevent neuronal death in vitro and in vivo, minimizing disease symptoms. The exosomes were able to improve the solubility and bioavailability of curcumin, increasing the penetration of the drug in the blood–brain barrier and preventing the death of neurons through the phosphorylation of the Tau protein through the activation of the AKT/GSK-3β pathway.

Furthermore, exosomes partake in organotropism, are bioavailable and have low toxicity and low immune responses [161]. As exosomes secreted by monocytes and macrophages can avoid entrapment in mononuclear phagocytes and, at the same time, improve drug delivery to target cells, they can be used to treat Parkinson’s disease. Thus, Haney et al. (2015) [162] developed exosomes loaded with catalase, which showed high encapsulation efficiency, sustained release, and preservation of catalase against protease degradation. The nanoparticles were readily absorbed by neuronal cells in vitro, and a considerable amount was also detected in the brains of mice with Parkinson’s after intranasal administration. The nanosystem provided significant neuroprotective effects, having the potential to be a versatile strategy in the treatment of inflammatory and neurodegenerative disorders.

#### 4.1.3. Nanostructured Lipid Carriers

Nanostructured lipid carriers are composed of a mixture of solid and liquid lipids, which results in a less organized lipid matrix with imperfections in the crystalline structure, which can lead to a greater accommodation of molecules. These nanoparticles share the advantages of traditional solid lipid nanoparticles, and the drug release profile can be easily modulated by varying the composition of the lipid matrix. Despite the presence of liquid lipids, the carrier’s matrix is solid at room and body temperature [163].

Nanostructured lipid carriers have the benefits of other carrier systems, such as physical stability, protection against degradation, and the controlled release of incorporated drugs (hydrophobic drugs are encapsulated, while hydrophilic ones can be dispersed on their surface). Additionally, this system has advantages over other nanoplatforms available for drug delivery, such as its easy scaling, absence of organic solvents in its composition (which guarantees greater safety), compatible constituents for parenteral administration, and low toxicity [164,165,166,167]. Compared to liposomes, for example, they guarantee greater stability of the encapsulated drug in addition to releasing the drug for a longer period due to its more crystalline structure [166].

One of the advantages of using these systems is that their nanometric size allows for better interaction with the stratum corneum, the outermost layer of the epidermis, which is made up of layers of dead cells. In addition, these carriers prevent the absorption of the active drug, forming an occlusive film that increases skin hydration by preventing water loss. They are even good candidates for encapsulating antioxidants, as they protect the molecule from degradation from light or oxygen, thus being indicated for developing topical drugs [168]. According to Li et al. (2018) [169], lipid carriers produced for the cotransport of lapachone and doxorubicin were able to overcome breast cancer multidrug resistance in MCF-7 tumor cell lines and tumor-bearing mice when compared to the same nanosystems carrying only one of the drugs or even the drugs in their free form.

Highly biocompatible nanostructured lipid carriers capable of co-administering paclitaxel and indocyanine for combined chemotherapy have also been successfully formulated. It was demonstrated in the study by Ding et al. (2017) [170] that the carriers could effectively protect the drugs and deliver them to tumor cells. They significantly increased drug stability, induced increased intracellular absorption of encapsulated drugs, and increased cytotoxicity in cancer cells thanks to the synergistic effects of co-administration. In hepatocellular carcinomas, different signaling pathways are deregulated, such as the epidermal growth factor receptor (EGFR) expression pathway. In the study proposed by Bondì et al. (2014) [171], a lipophilic low molecular weight EGFR inhibitor, which acts mainly on liver tumor cells, was encapsulated in nanostructured lipid carriers in order to overcome its low solubility and thus improve its anticancer activity.

Nanostructured lipid carriers, liposomes, and polymeric nanoparticles can also be modified to obtain reduced immunogenicity, greater bioavailability, and a better pharmacokinetic profile. One example is PEG coating for the parenteral administration of oridonine or docetaxel, which ensured greater drug targeting to the tumor site [172,173]. As there is a strong correlation between food and disease prevention, new technologies, including nanotechnology, are being introduced to enrich and produce more functional foods through isolation studies of their bioactive compounds [174]. The most important types of bioactive lipids that need to be supplied through food, for example, are fatty acids, carotenoids, antioxidants (such as tocopherols and polyphenols), and vitamins A and D, which are lipophilic [175]. In addition, omega-3s, the main essential fatty acids, are susceptible to oxidative deterioration; therefore, they require stabilization in an aqueous medium and protection. Thus, the encapsulation of omega-3 fatty acids appears as an alternative to reduce oxidation in fortified foods significantly [176]. Lipid carriers have encapsulated curcumin, quercetin, astaxanthin, vitamin C, vitamin A palmitate, α-lipoic acid, and green tea extract [177].

Essential oils are volatile constituents composed mainly of terpenes, which give aromatic plants their characteristic odor and provide pharmacological activities, including antitumor, antimicrobial, and anti-inflammatory. However, when administered, they have some difficulties regarding solubility in biological fluids and bioavailability due to their physicochemical properties. Therefore, delivering these oils to their targets is challenging, making their encapsulation in nanoparticles convenient [178].

To seek better forms of treatment for diabetes mellitus, Vieira et al. (2020) [179] proposed the production of nanostructured lipid carriers encapsulating sucupira essential oil using the high-pressure hot homogenization technique. Encapsulation efficiency was 99.98% with the optimized formulation following a modified release profile. In vitro cytotoxicity studies were also carried out in Caco-2 cells, demonstrating the nanosystem’s non-cytotoxic profile. The essential oil of Piper aduncum, an aromatic plant from the Amazon region with inherent anti-inflammatory activity, was encapsulated by Carneiro et al. (2022) [180] in nanostructured lipid carriers through the high-pressure homogenization technique to develop a cutaneous administration of its bioactive compounds. In the end, the particles obtained a size of around 130 nm, bioadhesive characteristics, and a controlled release profile with low irritant potential on the chorioallantoic membrane.

Another study sought to develop carriers containing pitavastatin together with the essential oil of the leaf of Pinus densiflora, which has antineoplastic properties, aiming to improve the treatment against oral squamous cell carcinoma, which is the most common epithelial tumor of the oral cavity. The optimized nanosystems had a particle size of 98 nm and a stability index of 89% with a synergistic effect between oil and pitavastatin in gingival cancer cells [181]. Furthermore, phospholipids, cholesterol, and PEGylated lipids may be added to these nanoparticles to improve their properties, including particle stability, biodistribution, safety, and delivery efficiency to the desired tissues or cells [182].

### 4.2. Polymeric Nanoparticles

Polymeric nanoparticles are generally biodegradable and biocompatible nanosystems that have high encapsulation efficiency, which are characteristics that make them great choices for drug delivery [183]. To be even more biocompatible, modified natural polymers have been used, such as synthetic polyesters and chitosan, which are non-toxic to the human body [184]. The diameter of polymeric nanoparticles varies between 20 and 1000 nm [139], and they are subdivided into vesicular systems (nanocapsules), in which the drug is encapsulated in a core surrounded by a polymeric membrane, and polymeric matrix systems (nanospheres), in which the drug is arranged throughout the polymeric matrix [184,185].

On the one hand, polymeric nanoparticles are promising vehicles for drugs, as they can be captured by cells and are well targeted. In addition, it is possible to control the release pattern of the encapsulated content through the chosen production technique, making them a promising alternative for the treatment of different types of cancer [184,186]. On the other hand, they have some disadvantages, such as low reproducibility, degradation problems, and potential antigenicity, even though the polymers used are natural [184].

Polylactic-co-glycolic acid (PLGA) nanoparticles, one of the most widely used types of polymers, have been approved by the FDA containing leuprolide acetate (a testosterone inhibitor) for use in the treatment of prostate cancer (Eligard^®^). The incorporation of PLGA allows a slow and sustained release of leuprolide acetate after subcutaneous injection administration [139]. Zhong et al. (2017) [187] encapsulated doxorubicin in hyaluronic acid nanoparticles and lipoic acid to treat multiple myeloma, resulting in a more prolonged and concentrated drug action at the tumor site, which helped protect the adjacent healthy tissues and organs.

Several vaccines and immunotherapies targeting B cell maturation antigen (BCMA) have been investigated to promote T cell activation for cancer treatment. A study proposed by Bae et al. (2020) [188] developed PLGA-based polymeric nanoparticles loaded with an immunogenic BCMA72-80 peptide. The result was a nanosystem successfully taken up by antigen-presenting cells, greater antitumor activity than the free peptide, and cytotoxicity due to degranulation and cytokines [188,189]. Another study of the antitumor activity of PLGA nanoparticles containing the programmed cell death ligand (PD-L1) was carried out by Guo et al. (2020) [60], resulting in a better activation of dendritic cells and T cells. Zuglianello et al. (2022) [190] produced nanoparticles by the association of pramlintide with dextran sulfate to serve as a new route of administration of peptides in the mucosa. The nanoparticles increased the α-helical content of pramlintide, stabilizing the peptide in its bioactive form. It was the first time that the feasibility of obtaining pramlintide–dextran polyelectrolyte nanoparticles with a high rate of drug encapsulation, nanometric size, and monodisperse particles was demonstrated, indicating their feasibility for the development of transmucosal delivery systems.

Another potential application of polymeric nanoparticles is against bacterial diseases. A study proposed by Valencia et al. (2021) [191] demonstrated that the production of this nanosystem associated with lecithin and chitosan encapsulating curcumin had a high encapsulation rate (92.74 ± 0.01%), high stability, and excellent antimicrobial activity against Gram-positive and Gram-negative bacteria, being able to preserve the antioxidant activity of curcumin. Quercetin, a bioactive compound with potential application as an antioxidant in food matrices, which has a high degree of hydrophobicity and low bioavailability, was also encapsulated in nanocapsules of lecithin and chitosan to improve its dispersibility in aqueous media and protect against degradation. An encapsulation efficiency of 98.31 ± 0.01% and stability of 28 d at 4 °C and 30 °C were achieved, making the nanoparticles excellent for potential application in food matrices aimed at developing new functional foods [192].

In addition, the polymeric nanoparticles can be associated with other materials, obtaining nanocomposites or hybrid to diverse applications [193,194]. Since significantly smaller quantities of raw materials are needed to obtain the nanomaterials, there is a sustainable behavior in the process [195,196]. With lighter and more resistant properties, these materials can reduce energy consumption and the resources needed for production, storage, and transport [195,197]. The reduced size and superficial area greater than its volume generate a positive impact on the environment by reducing their carbon footprint compared to their microscale homologs [8,198,199].

#### Polymeric Nanocapsules

Polymeric nanocapsules are commonly used as carriers for drugs whose oral administration would otherwise be considered pharmaceutically challenging. They can, for example, improve the oral bioavailability of drugs with low solubility in water (and, consequently, in biological fluids) and high molecular weight [200]. Also, by manipulating their physicochemical characteristics, such as adding PEG or chitosan, polymeric nanocapsules can be better directed to the desired targets and better released through the intestinal mucosa [201,202,203]. To reach specific targets, it is possible to manipulate polymeric nanocapsules by inserting, on their surfaces, specific ligands of the desired target, such as antibodies and peptides, which have specific cell receptors. Tumor cells themselves can express several molecular markers not expressed in normal tissues, likely becoming coupling sites for nanocapsules, making cancer treatment more specific [204].

Due to the success of PEGylation of proteins to improve systemic circulation time and decrease immunogenicity, one of the best examples of surface modifications for better absorption and targeting to tumors is the incorporation of PEG or polyethylene oxide, which prevents nanoparticles from being detected by the cells of the immune system as foreign objects, which allows them to circulate freely in the blood until they reach the tumor. Furthermore, PEG coatings also protect the surface against aggregation, opsonization, and phagocytosis, prolonging the systemic circulation time [205,206].

However, an excessive use of PEG can lead to several undesirable effects on the patient, since PEG tends to accumulate in body tissues [207]. Thus, polymeric nanocapsules can be developed by replacing this surfactant with more biocompatible ones when necessary. The use of poly(2-R-2-oxazoline), a class of polymers with a peptidomimetic structure belonging to the polyamide family, has been proven to be a promising alternative, as it presents cytocompatibility, hemocompatibility [208] and furtive behavior [137]. Almeida et al. (2019) [209] investigated the effect of the Cymbopogon citratus essential oil on nanocapsules under HaCat keratinocyte cells. Nanoparticles were able to control oil release and reduce its toxicity. Oregano oil was another essential oil successfully encapsulated in chitosan nanoparticles. The encapsulation took place through a method that produced an oil-in-water emulsion and ionic gelation of chitosan with sodium tripolyphosphate. In vitro assays showed a slow-release profile of the drug, which would lead to an increase in its bioavailability [210].

PEG-coated polymeric nanoparticles have also been produced to encapsulate garlic essential oil. Its insecticidal activity against the beetle Tribolium castaneum was evaluated. The encapsulated oil generated particles with less than 240 nm and more than 80% encapsulation efficiency, with the anti-insecticide action of the encapsulated oil being increased by seven times and maintained for five months, while the effectiveness of the free oil at the same concentration was only 11% [211].

Copaiba essential oil was another natural oil successfully encapsulated by Xavier-Junior et al. (2018) [212] in poly(isobutyl cyanoacrylate) nanocapsules through an original method of interfacial polymerization using chitosan as a stabilizer. The optimized nanocapsules showed a diameter of 473 nm, zeta potential of +34 mV, and oil encapsulation efficiency of 74%, including 55.5 μg of β-caryophyllene/mg of nanocapsules. In a subsequent study, Xavier-Jr et al. (2019) [213] investigated the mucoadhesive properties of paclitaxel-loaded chitosan-poly (isobutyl cyanoacrylate) core–shell nanocapsules for oral drug delivery. Nanoparticles presented a hydrodynamic diameter of 470 nm with a low polydispersity index, drug-loading capacity of 74 ± 1%, and high stability in the simulated gastric medium for 120 min and in storage at 4 °C for six months. These results make them promising oral delivery systems for anticancer molecules and bioactive compounds from the essential oil with a probable synergic effect.

Furthermore, Ivermectin, an antiparasitic widely used in medicine, was also encapsulated in nanocapsules of poly(ε-caprolactone) and pumpkin seed oil to increase its low oral bioavailability. The nanocapsules showed high encapsulation efficiency (98–100%) and stability at 4 and 25 °C for 150 d in addition to greater anthelmintic activity than the free drug. It also showed reduced toxicity against macrophages and fibroblasts [214]. Non-toxic and environmentally degradable polymers, such as polysaccharides (e.g., chitosan and hyaluronic acid), alginate, dextran, collagen, or synthetic polymers that have received FDA approval, including poly (lactide-co-glycolic acid) (PLGA) and poly(ε-caprolactone) are then advisable to maximize the safety of this type of nanoparticles [182].

### 4.3. Metallic Nanoparticles

Inorganic nanoparticles have been recently studied for drug delivery. In general, they consist of two areas: a core containing the inorganic component (e.g., gold, silver, or iron oxide) and a region that surrounds it, usually composed of organic polymers, which will mediate the interactions with the desired physiological components, protecting the material disposed of the core and directing it to the appropriate location [134].

Metallic nanoparticles have high stability, purity, optical and electromagnetic properties and are easily susceptible to surface modifications. Furthermore, when metal oxides are made into nanoparticles, they can have excellent photoluminescence, antibacterial, and antifungal properties, which are not always present in their macro forms [215]. The US Food and Drug Administration and the International Agency for Research on Cancer consider zinc oxide (ZnO) and titanium oxide (TiO_2_) to be “GRAS” (generally recognized as safe) [216].

Iron oxide nanoparticles also have super magnetic properties and can be produced by associating a biocompatible polymer with a Fe_3_O_4_ or Fe_2_O_3_ core. In addition to their application in targeted drug and gene delivery, iron oxide nanoparticles can be used in biosensors and magnetic fluid hyperthermia, which is a recent cancer treatment technique [217,218,219]. Zhang et al. (2015) [220] produced magnetic iron oxide nanoparticles with modified dimercaptosuccinic acid. Both bortezomib (BTZ), a first-line proteasome inhibitor, and gambogic acid (GA) have been encapsulated to enhance their anticancer properties. Combining BTZ and GA with iron nanoparticles resulted in a greater inhibition of cell proliferation and greater induction of cell apoptosis compared to the same free drugs at an equivalent dose [220]. Fe_3_O_4_ nanoparticles loaded with paclitaxel also have been synthesized to increase the low solubility of drugs in water, biological fluids, and most excipients used by the pharmaceutical industry. Paclitaxel is one of the most effective antineoplastic drugs identified in recent decades mainly because it has demonstrated action against a broad spectrum of cancers, such as ovarian, breast, glioma, and multiple myeloma. Results have shown excellent stability and a greater inhibition of tumor volume of nanoparticles when compared to the free drug with clear inhibitory effects on tumor growth in animal models [221,222].

Gold and silver nanoparticles are, on the other hand, the most common metallic nanoparticles used as drug carriers and have become the subject of intensive research [223]. Depending on the size presented, gold nanoparticles can be used to diagnose and treat cancer [224,225]. Acute cytotoxicity data suggest surface-functionalized gold nanoparticles are not inherently toxic to healthy human cells. However, nanoparticles with a diameter <2.0 nm have shown high toxicity due to their ability to cross the nuclear pores and enter the nucleus. Gold nanoparticles larger than 10 nm are characterized by lower cytotoxicity [226]. Then, it is still necessary to study its complete toxicity profile, especially in the long term [227], even though their mechanism of action is cellular uptake. In addition, gold nanoparticles pose significant environmental challenges, including the potential release of metal ions. The structural modification of gold nanoparticles provides opportunities for high-quality production, thereby mitigating concerns about their environmental toxicity [228].

When loaded with oligonucleotides, the resulting gold nanoparticles are called spherical nucleic acids [229], which are nanostructures with a high capacity for internalization by various types of cells but presenting low toxicity in healthy cells. Nanoparticles functionalized with oligonucleotides, such as siRNAs, can be used in different applications related to the regulation of genes or immunomodulatory processes, as described for the treatment of diseases such as breast cancer [230], glioblastoma [229], psoriasis and diabetes [231].

Silver nanoparticles, on the other hand, can be used as biosensors due to their optical properties and ability to absorb and scatter light [232,233] as well as for drug delivery, which is mainly due to their ability to conjugate with antibodies, ligands, and drugs [234]. It was discovered that silver nanoparticles induce cytotoxicity through the apoptosis and necrosis of several cells, including tumor cells, in addition to inhibiting or decreasing DNA damage, the generation of reactive oxygen species, and the inhibition of stem cell differentiation [235]. Nanoparticles can be used to deliver a combination of drugs, including salinomycin, gemcitabine, and camptothecin [236].

Gemcitabine encapsulated in silver nanoparticles demonstrated a synergistic effect by generating more cytotoxicity and apoptosis in ovarian cancer cells than when using the drug in its free form, as shown by Yuan et al. (2017) [237]. In addition, it was noted that they can improve the responsiveness to gemcitabine in the same cancer cells, which consequently leads to an increased production of pro-apoptotic genes and the activation of caspases 3 and 9 [236,237].

Silver nanoparticles have also been used as antiviral agents due to their inhibitory activity against several types of viruses, such as some coronavirus strains, hepatitis, influenza, herpes, and HIV, among others [238,239,240]. They contribute to viral inactivation due to reactions with sulfide, amino, carboxyl, phosphate, and imidazole groups [241] in addition to blocking viral entry into the cell and, consequently, preventing infection [242]. The positive results related to the prevention of H_3_N_2_ influenza virus infection by silver nanoparticles have been shown in both in vitro and in vivo studies, which confirmed that the growth of the virus hemagglutinin activity is inhibited as the dose increases [240].

In addition, silver nanoparticles can also be designed to increase the efficacy, stability, specificity, and biocompatibility of antibiotics [243] through different mechanisms of action, many of which are based on adherence to microbial cells as well as their penetration, the generation of reactive oxygen species and the modulation of transduction pathways [244]. Because they have such a small diameter, they can penetrate bacterial cells to generate an inhibition of the enzymatic systems of the respiratory chain to impair DNA synthesis [245].

As shown in this section, nanoparticles from different sources, whether organic or inorganic, lipids or polymers, have numerous advantages compared to the usual pharmaceutical vehicles, being recently deeply studied either for the improved delivery of drugs already used in clinical practice or for those that have not yet reached the market. Thus, technological nanosystems are extremely promising for treating various diseases, including those previously incurable, such as cancer. Given the numerous articles published in recent years on nanotechnology in drug delivery, we decided to simplify access to this knowledge by compiling several studies into a comprehensive table, aiming to provide a quick overview of the various types of nanomaterials and their therapeutic applications (Table 1).

## 5. Nanomaterials for Imaging and Diagnosis

As molecular imaging has improved biomedical imaging, new tools have been developed to address clinical needs such as disease staging, stratification, and the monitoring of treatment response [246]. Nanomaterials, as defined by the International Organization for Standardization (ISO) (ISO, 2023), have been proposed as new candidates for imaging and diagnosis tools in various ways depending on their properties and modifications. Nanoparticles can be introduced into the body in a noninvasive manner, and when skillfully engineered, they can attain tissue specificity by utilizing targeting components [247]. These systems possess an inherent multifunctional and modular nature, allowing them to facilitate tissue targeting and selectivity, and the chemistry of nanoparticles can be adjusted to influence factors such as circulation half-life [205,248,249].

**Figure 4:**
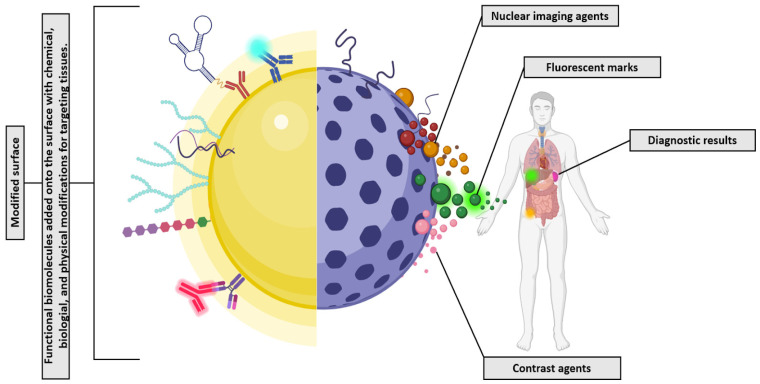
Functionalized nanoparticles and nanosystems for bioimaging and diagnostic purposes.

It is possible to adjust and expand the surface functionalization of nanomaterials in terms of their physical, chemical, and biological properties (Figure 4). Multifunctional nanosystems can be modified by exploiting surface functionalization in one of three ways: (i) by adding new functional molecules to the surface, (ii) by covering the surface with another layer, or (iii) by modifying their chemical termination directly on the surface [250,251]. When modified on the surface, the new nanoplatforms exhibit multiple actions, combining therapy and diagnosis to produce a theragnostic response [252,253]. As well as protecting drugs from degradation and controlling their bioavailability and release, functional coatings prevent the degradation of drugs or bioactive agents [254].

Theragnostic applications use functionalization as a powerful technique to use nanoparticles in the medical field. The advantage of this approach is that it can enhance therapeutic systems’ versatility and widen the range of bioimaging modalities to be used [255]. The integration of therapeutic functionality and imaging capabilities will offer benefits for monitoring drug delivery effectiveness, detecting potential off-target effects and toxicity, and observing the therapeutic response. The result has been the development of a broad range of nanotherapeutic agents, including liposomes, polymeric nanoparticles, and coated metal nanoparticles, for various imaging-guided techniques. There are various molecular imaging techniques available to diagnose diseases, including optical imaging, Magnetic Resonance Imaging (MRI), Positron Emission Tomography (PET), and Single Photon Emission Computed Tomography (SPECT), though their sensitivity and resolution differ [23,246,256,257,258,259,260]. In general, nanotherapeutic agents can be modified by targeting moieties to enhance photoluminescent effects for optical imaging [261]. Additionally, 2D nanomaterials can be used for MRI, which provides various information related to living subjects’ anatomical, physiological, and molecular processes [23,262].

As a result of inherent limitations (photobleaching or poor contrast generation), organic and organometallic imaging agents were often used as imaging agents. Several classes of inorganic nanoparticles have been developed to overcome these limitations in clinically relevant imaging modalities [263]. As diverse ligands can envelop nanoparticles, they exhibit robust affinity and distinctiveness, promoting interaction with specific sets of target cells. This attribute enables a notable enhancement in their enduring association, presenting an escalation of four to five orders of magnitude [263,264]. Most nanoscale imaging agents typically exceed 10 nm in size, evading renal elimination and prolonging their circulation time compared to smaller counterparts, spanning minutes versus days [249,265]. This advantageous trait facilitates iterative image scrutiny, obviating the requirement for additional nanoparticle administration [266].

The contrast in the MRI is generated based on variances in the density of hydrogen protons along with the longitudinal T1 (spin–lattice relaxation time) and transverse T2 (spin–spin relaxation time) relaxation characteristics of protons within the soft tissue [267]. Thus, it has been demonstrated by J. Y. Park et al. (2009) [268] that ultra-small gadolinium oxide (Gd2O3) nanoparticles with a relaxivity value of 9.9 nM^−1^s^−1^ can be used in clinical imaging as T1-weighted MRI agents. By increasing the particle size from 4 to 12 nanometers, Jun et al. (2005) [269] have developed Fe_3_O_4_ nanocrystals as size-dependent T2-weighted MRI agents. Tong et al. (2010) [270] exhibited that superparamagnetic iron oxide nanoparticles (SPIONs) measuring 14 nm in diameter, which were enveloped with hydrophilic polymer polyethylene glycol-phospholipids, exhibited a remarkable 200-fold increase in T2 relaxivity (385 ± 39 mM^−1^s^−1^) in comparison to the uncoated Fe_3_O_4_ particles.

Wang et al. (2013) [271] have created hybrid nanocomposites by incorporating gadolinium-doped layered double hydroxide (LDH) and gold (Au) combined with the anti-cancer medication doxorubicin (DOX). These nanocomposites exhibited a longitudinal relaxivity value of 6.6 mM^−1^s^−1^. Importantly, these nanocomposites displayed minimal cytotoxic effects when tested against both normal fibroblast (L929) and cervical cancer (HeLa) cells. Furthermore, 2D nanomaterials have the potential to serve as diagnostic agents by integrating radioisotopes like 57Co, 64Cu, 67Ga, and 68Ga into their crystal lattice. This is facilitated by their chemical stability and minimal toxicity within in vivo systems [272,273,274].

Through nanotechnology, experimental data visualization can be enhanced through optical imaging, enabling heightened accuracy and resolution both in space and time. An approach to this concept relies on functionalized semiconductor nanocrystals, which are also known as quantum dots. Unlike bulk materials, nanoscale particles exhibit distinct quantum mechanical properties encompassing electrical, thermal, and optical characteristics. An integral component of these devices is heavy metals (cadmium–selenium or cadmium telluride), an unreactive zinc sulfide shell, and external coatings that can be systematically engineered to fulfill specific functional needs using specialized bioactive compounds on their surfaces [275]. It has already been extensively demonstrated that quantum dot labeling can enable single particle tracking within live cells in vitro across a wide range of cell types [276].

Employing nanomaterials for optical imaging also presents a promising avenue for the enhanced visualization of cerebral injuries, such as traumatic brain injury. In this context, necrotic brain cells were selectively targeted through the application of PEGylated poly(lactic-co-glycolic acid) (PLGA) nanoparticles, which encapsulated both perfluorocarbons and near-infrared fluorophores. Using cyanine dyes, including IRDye 800CW, these nanoparticles were tracked by optical imaging and 19-fluorine MRI, presenting accumulation in blood regions for prolonged periods, effectively homing in on traumatic brain injury-damaged tissue. A significant advantage of these necrosis-targeting nanoparticles is that they can provide quantitative three-dimensional insights into deeper tissues via MRI as well as rapid qualitative optical monitoring of TBI, making them excellent candidates for clinical diagnosis and assessment [277].

Within the realm of varied nanomaterial advancements, metal-based inorganic nanoparticles possessing elevated atomic numbers and substantial X-ray attenuation coefficients exhibit significant promise in enabling precise bioimaging applications, serving as contrast agents in computed tomography [278]. Gold nanoparticles (AuNPs) demonstrate remarkable photothermal characteristics, making them potential alternatives to lasers [279]. These AuNPs possess the capability to absorb light within the near-infrared spectrum and subsequently transform this energy into heat. The photothermal attributes of AuNPs have been extensively examined through both in vitro and in vivo investigations, involving the scrutiny of glioma cells and mouse models [280]. These materials possess notable X-ray attenuation capabilities and a substantial K-edge energy (80.7 keV) with the potential to deliver enhanced imaging contrast when compared to iodinated contrast agents at equivalent concentrations [281]. Gold nanoparticles (4 nm and 15 nm) generated a more persistent and pronounced contrast within blood vessels compared to their larger counterparts. This heightened contrast arises from the diminished recognition and clearance of smaller material by the mononuclear phagocyte system as they navigate the liver and kidney, ultimately leading to an elevated concentration of nanoparticles within the blood pool [282]. Engineered gold nanorod (AuNR) nanoprobes, modified with Arg-Gly-Asp (RGD), exhibited attributes of non-toxicity, substantial contrast enhancement, and extended imaging duration [283].

Another method worthy of consideration is the exploration of photoacoustic imaging (PAI), which furnishes molecular insights by analyzing the multispectral photoacoustic (PA) reactions within biological tissues [284]. Consequently, it is applicable for functional imaging purposes, including the tracking of hemoglobin oxygen saturation levels (sO2) [285], the examination of melanin constituents [286,287,288], and detecting lipids [289].

It is important to remember that nanomaterials, despite their small scale, possess unique physicochemical properties, which allow them to form nanostructures with greater surface-to-volume ratios compared to most nanomaterials. This ample surface area allows the attachment, absorption, and transport of various molecules like small-molecule drugs, probes, RNA, DNA, and proteins by nanostructures. As a result of their adjustable size, surface features, and structural configurations, nanomaterials have significant durability, significant capacity, inherent hydrophilic and hydrophobic qualities, and versatile delivery methods. Due to these attributes, nanomaterials are highly sought after in a wide range of medical fields [249,290]. Table 2 represents some notable studies exemplifying these endeavors in the development of technology to diagnosis.

## 6. Biological Risks and Biocompatibility of Nanomaterials

Nanotechnology brings numerous benefits to the health field, which is mainly due to the physicochemical properties of nanomaterials. But the nanoparticles may also have completely different characteristics from macroparticles, including unfavorable ones [216]. There are concerns associated with the accumulation of nanomaterials in the body [306] and its consequent toxicity in vivo [307,308] due to the reactivity of nanomaterials on biological tissues [309]. Some routes of exposure are inhalation, oral, topical, and intravenous. This can lead to agglomeration/aggregation, dissolution, and the formation/evolution of the corona, among others, affecting the toxicokinetics and biological effects of nanomaterials in the body [182]. Thus, the surface area, size, stability, and solubility in water, among others [290], are some factors that need to be investigated to ensure the safety of nanomaterials. Barreto et al. (2019) [310] showed that the bioaccumulation and impact of AuNPs were influenced by the nanoparticle size, surface coating, surface charge, and state of aggregation or agglomeration. Indeed, the size is a crucial factor for these nanosystems. Due to their small size, cellular interactions become greater. Thus, nanoparticles can interact with the cell membrane surface, acting as ligands to the receptors in this region, allowing for more cellular interaction [311]. In addition, the reduced size of nanoparticles facilitates the penetration of nanomaterials in some cell types, such as endothelial, pulmonary epithelia, intestinal epithelium, alveolar macrophages, neuronal cells, and other macrophages, through cellular diffusion [312]. As a consequence, genotoxicity, inflammation, oxidative stress, apoptosis, and necrosis can occur, leading to numerous complications, such as fibrosis, carcinogenesis, and cardiovascular problems [313]. Furthermore, nanomaterials (NMs) can cause defects or changes in DNA [314] and inflammation through the release of cytokines and chemokines [315]. Oxidative stress caused by NMs can also lead to defects in cell signaling [316].

It is also important to highlight that the accumulation of NMs in the intracellular environment facilitates their release into the bloodstream and can be transported to other organs, including the liver [315], kidneys [317], spleen [318], lymph nodes [319], heart [320] and even the brain [321]. Nanomaterials can still cause damage in locations further away than the exposure site, significantly increasing the risk in the long term. The respiratory system, gastrointestinal tract, and contact with the skin [306] are some means of exposure since the organs are directly in contact with the external environment.

In the respiratory system, the mechanism of toxicity occurs primarily through cellular diffusion, where tiny particles of nanomaterials enter the cell through this means of transport. According to Thu et al. (2023) [306], the nanotoxicity of this system occurs when nanoparticles interact undesirably with respiratory tissues due to free radicals, producing oxidative stress. Consequently, unwanted complications may appear, such as cell membrane damage, lysosomal damage, DNA deterioration, mitochondrial damage, etc.

The liver, in turn, is the organ most vulnerable to nanotoxicity from nanomaterials since ultrafine particles can be deposited in the liver and accumulate [317] over a long period [322]. As the organ responsible for the detoxification of harmful chemicals present in the system [313], the liver plays a crucial role in synthesizing proteins, hormones, and metabolism. Any damage to this organ can lead to several complications, such as multiple organ failure, the accumulation of toxic substances, dysregulation in the immune system, and the poor absorption of nutrients, among others.

The kidneys are next in line as the organs most susceptible to the accumulation of nanoparticles. They are responsible for blood filtration, fluid regulation, mineral regulation, and blood pH, among other functions [323]. Thus, if NMs are present in the bloodstream, they will be sent to the kidneys for filtration. Therefore, nanoparticles in this organ can also cause a series of complications, among the main ones nephrotoxicity and kidney damage [324].

The shape of nanomaterials is another parameter that influences the interaction with biological systems; that is, it is directly related to the absorption of nanoparticles in the cellular environment, causing toxicity [325]. Still, the surface chemistry of the nanomaterial can alter its affinity with water and generate aggregations [326].

**Figure 5:**
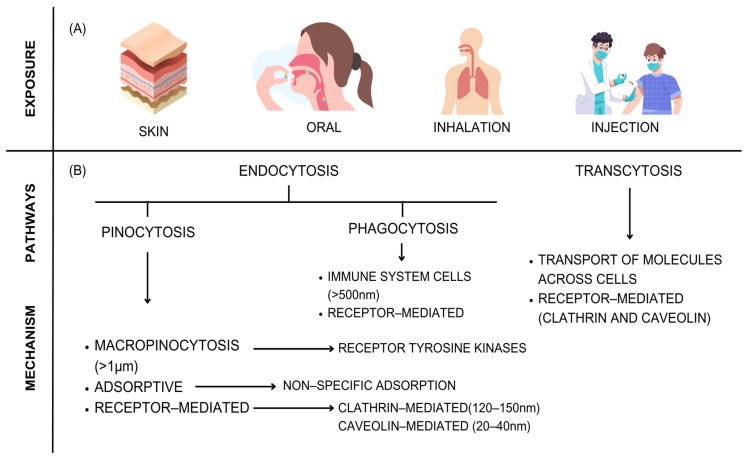
Forms of exposure to nanomaterials (**A**), their entry methods, and mechanisms in the intracellular environment (**B**).

Figure 5 shows the forms of exposure, entry methods, and mechanisms in the intracellular environment of nanomaterials. NMs can be dispersed to other tissues through endocytosis [327], phagocytosis [328], pinocytosis [329], and transcytosis [330] (movement between epithelial and endothelial cells).

Endocytosis encompasses large particles present in extracellular fluid [331] and can be divided into two types: phagocytosis and pinocytosis. Phagocytosis occurs in specialized cells composed of immune system cells (neutrophils, macrophages, dendritic cells, and monocytes) and encompasses particles larger than 500 nm through a receptor-mediated process [332]. Proteins (opsonins) then mark the nanoparticles still in the bloodstream and make them visible to macrophages [333]. On the other hand, pinocytosis occurs through three different mechanisms: macropinocytosis, adsorbent-mediated pinocytosis, and receptor-mediated pinocytosis. In general, in pinocytosis, cellular uptake occurs in the extracellular fluid. Thus, macropinocytosis is cellular uptake in a nonspecific manner (particles > 1 μm) and is initiated by the stimulation of some growth factors, such as tyrosine kinase [334]. Adsorbent pinocytosis is the nonspecific binding of solutes to the cell membrane (>200 nm); that is, NMs bind nonspecifically to some complementary binding sites on the cell surface [329] such as the binding of cationic particles to a negatively charged cell surface. Finally, receptor-mediated pinocytosis (mediated by claritin and caveolin) is highly selective and specific. The claritin-mediated mechanism allows the passage of particles (between 120 and 150 nm) in claritin-coated vesicles [335]. Caveolin-mediated pinocytosis enables the selection of 20–40 nm particles [336].

In addition, transcytosis is a process in which receptors and ligands are transported from one side of the cell to the opposite location. In other words, biomacromolecules are transported through biological barriers within one or more membrane-bound transporters. This mechanism is one of the main dispersers of nanomaterials within the organism, such as the transcytosis of nanoparticles from the epithelium of the respiratory tract into the blood circulation or lymphatic vessels [337,338,339,340]. This process is usually mediated by receptors such as claritin and caveolin.

Some strategies have been developed to mitigate the adverse effects and possible toxicity of nanosystems, such as manipulating and modifying the physical–chemical composition of their surfaces. An example is the coating of silica or biodegradable polymers to control the disintegration of particles and the release of kinetics of metal ions. Another strategy would be the development of nanoparticles in cellular plasma membranes, such as membranes derived from erythrocytes, which generate minimal protein corona formation, reducing the toxicity and immunogenicity caused [341,342]. Iron oxide nanoparticle (IONP) toxicity can also be modulated and mitigated by choosing potential use pathways, as shown by [343].

It is also important to emphasize the biocompatibility of nanomaterials, which differs from toxicity. Toxicity encompasses the adverse effects caused by substances in living organisms. Biocompatibility is the ability of a material to perform its function in the intracellular environment without causing undesirable reactions [344]. In other words, when a foreign body encounters the human body, it generates a response. If this response is undesirable, the material becomes incompatible and toxic. The biocompatibility of nanomaterials is highly dependent on their surface properties, size, shape, functional groups, concentration, and dosage [345]. According to the Registration, Evaluation, Authorization, and Restriction of Chemical Substances (REACH), the nanomaterials must meet the safety standards adopted for conventional chemicals, such as evaluation effects, exposure, and risk characterization [346].

In vivo testing is crucial to demonstrate the safety and biocompatibility of nanomaterials. The type of nanomaterial and its application directly determine the type of animal used, the drugs, the administration, the dosage, and the other parameters used [347]. In addition, there is a safety protocol to affirm the biosafety of nanomaterials and associated devices: ISO/TR 10993-22:2017 part 22 [348]. This document informs the necessary tests for nanomaterials, such as (i) cytotoxicity, (ii) genotoxicity, carcinogenicity, and reproductive toxicity, (iii) immunotoxicity, irritation, and sensitization, (iv) hemocompatibility, (v) systemic toxicity, (vi) pyrogenicity, and (vii) implantation [349]. Therefore, the challenge of approving nanosystems for biomedical engineering applications requires an assessment of fate and toxicity accomplished through different pre-clinical and clinical phases approved by specific regulatory agencies.

## 7. Conclusions

The intersection of nanotechnology with biomedical engineering has generated significant health impacts with advances in diagnostics and imaging, nano-biosensor development, controlled drug release, and nanomaterials in regenerative therapies, among others. The possibility of directing specific target cell therapies and detecting biomarkers in the initial stages is an example of how this synergy between areas has favored medicine. Drug-controlled release systems have expanded therapeutic options and improved the effectiveness of treatments. Nanofibers and nanowires have a central role as scaffolds in tissue engineering. The high ratio of surface area/volume, flexibility, and porosity create conducive environments for cellular regeneration and interaction, assisting in treating injuries and diseases. Nanomaterials for images and diagnosis also open a remarkable boundary in biomedical engineering. Its intrinsic versatility and multifunctionality, directed specificity, lasting image patterns, and the ability to cross barriers previously considered insurmountable offer unprecedented accuracy and insight into the complexity of the human body. However, there are still challenges to overcome in the nanotechnology and biomedical engineering connection, such as the stability and uniformity of nano-biosensor functionalization; biomimetic scaffolds, according to the biological complexity of tissues; nanomaterial production scale; and security guarantee, potential toxicity, and long-term biocompatibility. Given this, research should continue to investigate the influence of nanoparticle structure and diameter, preparation methods, surface modifications, and, especially, the interaction of nanomaterials with organs and tissues, directing target application. Nanotoxicological and sustainable use studies of nanomaterials are essential to assess their safety and regulation, including evaluations of their bioavailability, and biological impacts. Therefore, the potential of these technologies is immense, but it must be explored with a multidisciplinary perspective in medicine. Including professionals from various areas will assist in developing reproducible and viable safe devices for the market and society.

## Figures and Tables

**Table 1:** Main nanosystems and some of their applications in drug delivery.

Nanosystem	Composition	Therapeutic Application	Characterization	Reference
Liposome	PEG-coated liposomal doxorubicin	Treatment of breast cancer	—	[134]
	Liposome system carrying prostaglandin E-1 (PGE-1)	Treatment of cardiovascular diseases	—	[146]
	Liposome modified with pyrrolidinium surfactants containing a hydroxyethyl fragment	Transdermal delivery of non-steroidal anti-inflammatory drugs	—	[149]
	Liposome coated with PEG	Ischemic myocardium	—	[147]
Exosome	Exosomes by bovine milk	Treatment of lung tumor	Size < 80 nm; PDI of 0.22 ± 0.06	[155]
	Exosomal formulations	Treatment of cancer	—	[157]
	Exosomes derived from adipose tissue-derived mesenchymal stem cells	Treatment of type 1 diabetes mellitus.	Size ≤ 100; diameter of 41.1 nm	[159]
	Exosomes containing curcumin	Treatment of Alzheimer’s disease	—	[160]
Nanostructured lipid carrier	Nanostructured lipid carriers produced for cotransport of lapachone and doxorubicin	Treatment of breast cancer	—	[169]
	Nanostructured lipid carriers encapsulated with lipophilic low molecular weight EGFR inhibitor	Hepatocellular carcinomas	—	[171]
	Nanostructured lipid carriers with omega-3 fatty acids	Reduce oxidation in fortified foods	—	[176]
	Nanostructured lipid carriers encapsulating sucupira essential oil	Treatment for diabetes mellitus	Size 148.1 ± 0.9815 nm; PDI 0.274 ± 0.029; ZP from −0.00236 ± 0.147 mV	[179]
	Nanostructured lipid carriers encapsulating essential oil of Piper aduncum	Anti-inflammatory activity	—	[180]
	Nanostructured lipid carriers encapsulating essential oil of the leaf of Pinus densiflora	Treatment against oral squamous cell carcinoma	72 ± 1.5 and 120 ± 5.1 nm; PDI 0.1 to 0.29; stability index of 89%	[181]
Polymeric nanoparticle	Polylactic-co-glycolic acid (PLGA) nanoparticles	Treatment of prostate cancer	—	[139]
	Nanoparticles of hyaluronic acid and lipoic acid encapsulated with doxorubicin	Treatment of multiple myeloma	Size of 183 nm	[187]
	PLGA-based polymeric nanoparticles loaded with an immunogenic BCMA72-80 peptide	Treatment of cancer	Size 172 ± 0.73 nm; PDI of 0.20 ± 0.01; Zeta potential of −1.16 ± 0.18 mV	[188]
	PLGA nanoparticles containing the programmed cell death ligand (PD-L1)	Activation of dendritic cells and T cells	—	[60]
Iron oxide nanoparticle	Magnetic iron oxide nanoparticles with modified dimercaptosuccinic acid	Inhibition of cell proliferation and greater induction of cell apoptosis	Diameter of 18.7 ± 5.61 nm	[220]
	Fe_3_O_4_ nanoparticles loaded with paclitaxel	Treatment of tumor	—	[221]
Gold nanoparticle	Gold nanoparticles loaded with oligonucleotides	Regulation of genes or immunomodulatory processes	—	[229]
	Gold nanoparticles	Treatment of cancer	Size 4 nm	[224]
Silver nanoparticle	Gemcitabine encapsulated in silver nanoparticles	Ovarian cancer cells	Size 20 nm; PDI 0.123; Zeta potential −33 mV;	[237]
	Silver nanoparticles	Prevention of H3N2 influenza virus	Diameter of around 9.5 ± 0.8 nm; stable over 6 months at room temperature	[240]

**Table 2:** Nanosystems formulated to improve the diagnosis of several diseases.

Nanosystem	Characteristics	Application	Main Results	Reference
Bismuth oxide nanoparticles (HA-Bi_2_O_3_)	-	Bismuth oxide nanoparticles	Exhibit great promise for CT imaging-guided radiotherapy in the diagnosis and treatment of tumors	[291]
Perfluorocarbon liposomes with gold nanospheres	Size: 200 nm; light-to-heat conversion effect under 808 nm by NIR laser irradiation	Ultrasound imaging-guided photothermal chemotherapy	A remarkably enhanced ultrasound signal was detected. Exhibited a prominent photothermally reinforced chemotherapeutic effect	[292]
Nanobubble-paclitaxel liposome (NB-PTXLp) complexes	Entrapment efficiency of 85.4 ± 4.39% Conjugation efficiency ~98.7 ± 0.14%) Size: 200 nm	Ultrasound imaging and ultrasound-responsive drug delivery in cancer cells	The developed nanobubbles were found to exhibit more than 1-week echogenic stability as opposed to 6 h stability of the commercially available ultrasound contrast agent SonoVue	[293]
Magnetic-carbon-quantum-dots-probe-labeled apoferritin nanocages	Average diameters of ~5 nm Polydispersity index of Gd-CDs was 0.201	Bioimaging and targeted therapy	Unique green photoluminescence and almost no toxicity Could serve as an excellent T1 contrast agent for MRI	[41]
Deoxyglucose-conjugated persistent luminescent nanoparticles	Hydrodynamic diameter: 500 nm. Zeta potential was about +1 mV	Diagnostic application in fibrosarcoma tumor model	Significant accumulation of nanoparticles to the tumor site. They showed a higher killing ability for cancer cells compared to normal cells	[294]
Functionalized upconverting nanoparticles (UCNPs)	Particle size of 29.0 ± 0.4 nm. Ligands added to the surface: 32 nm for ligand-free UCNPs, to 50 nm for EY-UCNPs, and 100 nm for the EY-PEG-UCNP, with polydispersity indexes of 0.6, 1.2, and 0.4, respectively	Nanophotosensitizers and deep tissue bioimaging agents for simultaneous therapeutic and diagnostic applications	The functionalized UCNPs present deep tissue NIR-II fluorescence under 808 nm excitation, thus demonstrating their potential as bioimaging agents in the NIR-II biological window	[295]
Multicore magnetic iron oxide nanoparticles	Low hydrodynamic size of 35 nm, with good monodispersity, PDI < 0.13. Stability over at least 6 months	Mediators for AC-magnetic field hyperthermia and as contrast agents for MRI	Potential application in MRI and magnetic hyperthermia. PEG can stabilize the nanoparticles, which can improve deposition. As well, there is potential for suspensions as T2-contrast agents with good bio-stability and persistent magnetic responses following uptake, which can mark liposome deposition and may provide local hyperthermic hot spots	[296]
Gold nanoparticles radiolabeled with 99mTc.	Au-citrate NPs with 13.8 ± 1.2 nm Au-MUAM-PADA ** NPs with 14.0 ± 0.9 nm, Au-MUAM-PADA-99mTc(CO)_3_ NPs with 13.9 ± 1.2 nm	Theragnostic applications	The multifunctional Au NPs could be used for targeted drug delivery and imaging of cancer cells as well as for monitoring the effectiveness of treatment	[297]
Gold nanorods	Particle sizes of 177.9 ± 19.3 nm for eAuNR (PDI 0.46 ± 0.14) and 149.5 ± 9 nm for mAuNR (PDI 0.32 ±0.07) ***	Nanomaterials for target delivery	Important delivery differences exhibited by extracellular vesicles (EVs) or cell membranes- coated nanorods, an understanding of which may be important to the design and development of nanomaterials that use these coatings for target and delivery	[298]
rGO-AuNPs- PEG *	Size: 160 nm	Photothermal agent	Very strong SERS signal, NIR-II PA signal and high photothermal efficiency against tumor upon 1061 nm laser irradiation	[299]
Lanthanide-activated nanoparticles	-	Biological application: bioimaging, X-ray imaging, MRI, oncotherapy, photodynamic therapy, etc.	Nanosystems with multiple modalities of bioimaging, oncotherapy, and neuromodulation	[300,301]
Core/shell lead sulfide/cadmium sulfide (CdS) quantum dots (CSQDs)	Core size to ~5.4 nm for ~1600 nm emission, forming an ~1.5 nm thick CdS shell	Bright fluorescent probe emitting at ~1600 nm in the NIR	Exhibited long blood circulation time, real-time imaging, CSQDs are excreted through the biliary pathway without toxicity effects	[302]
SPIONs (iron-oxide NPs)	Surface modification 1,5-dihydroxy-1,5,5-tris-phosphonopentyl-phosphonic acid (di-HMBPs)	Osteoporosis	High affinity for calcium ions/hydroxyapatite for MRI use	[303]
Carbon nanotubes	99mTc-labeled carbon nanotubes	Active bone metabolism	High affinity for hydroxyapatite for photoacoustic imaging use	[304]
Quantum dots	Surface modification with various antibodies	Binding to unique cell populations in bone marrow	Targeted cell imaging	[305]

* Gold nanoparticle-coated reduced graphene oxide functionalized with PEG. ** Picolylamine Diacetic Acid (PADA); Mercaptoundecylamine (MUAM); Surface-Enhanced Raman Scattering (SERS), Second Near Infrared (NIR-II); Quantum Dot (QD); Commercial Quantum Dots (Qdots); Fetal Bovine Serum (FBS); monoclonal Antibody (mAb) anti-mouse CD31; Antibody (Ab). The numbers 625 and 612 stand for wavelength in nanometers, and NB stands for norbornene. *** EVs/AuNRs (eAuNR), and cell membrane/AuNRs (mAuNR) systems.

## Data Availability

The data presented in this study are available on request from the corresponding author.
